# Characterization of tau oligomeric seeds in progressive supranuclear palsy

**DOI:** 10.1186/2051-5960-2-73

**Published:** 2014-06-14

**Authors:** Julia E Gerson, Urmi Sengupta, Cristian A Lasagna-Reeves, Marcos J Guerrero-Muñoz, Juan Troncoso, Rakez Kayed

**Affiliations:** Mitchell Center for Neurodegenerative Diseases, University of Texas Medical Branch, Galveston, TX 77555 USA; Departments of Neurology, Neuroscience and Cell Biology, University of Texas Medical Branch, Galveston, TX 77555 USA; Department of Neuropathology, Johns Hopkins University, Baltimore, MD 21205 USA

**Keywords:** Tau, Oligomers, Progressive supranuclear palsy, Seeding

## Abstract

**Background:**

Progressive supranuclear palsy (PSP) is a neurodegenerative tauopathy which is primarily defined by the deposition of tau into globose-type neurofibrillary tangles (NFT). Tau in its native form has important functions for microtubule dynamics. *Tau* undergoes alternative splicing in exons 2, 3, and 10 which results in six different isoforms. Products of splicing on exon 10 are the most prone to mutations. Three repeat (3R) and four repeat (4R) tau, like other disease-associated amyloids, can form oligomers which may then go on to further aggregate and form fibrils. Recent studies from our laboratory and others have provided evidence that tau oligomers, not NFTs, are the most toxic species in neurodegenerative tauopathies and seed the pathological spread of tau.

**Results:**

Analysis of PSP brain sections revealed globose-type NFTs, as well as both phosphorylated and unphosphorylated tau oligomers. Analysis of PSP brains via Western blot and ELISA revealed the presence of increased levels of tau oligomers compared to age-matched control brains. Oligomers were immunoprecipitated from PSP brain and were capable of seeding the oligomerization of both 3R and 4R tau isoforms.

**Conclusions:**

This is the first time tau oligomers have been characterized in PSP. These results indicate that tau oligomers are an important component of PSP pathology, along with NFTs. The ability of PSP brain-derived tau oligomers to seed 3R and 4R tau suggests that these oligomers represent the pathological species responsible for disease propagation and the presence of oligomers in a pure neurodegenerative tauopathy implies a common neuropathological process for tau seen in diseases with other amyloid proteins.

## Introduction

Progressive supranuclear palsy (PSP) is a common parkinsonian neurodegenerative tauopathy which is characterized by the development of dementia, changes to personality, visual and speech deficiencies, and gait alteration [1]. It affects about 6.5 in 100,000 people [2]. Pathologically, PSP is primarily defined by the deposition of tau into neurofibrillary tangles (NFT). Tau in its native form has important functions for microtubule stabilization and neurite growth. *Tau* undergoes alternative splicing in exons 2, 3, and 10 which results in six different isoforms of tau protein in the adult central nervous system [3]. Products of splicing on exon 10 are the most prone to mutations and result in three isoforms with three microtubule binding repeats (3R tau) and three isoforms with four microtubule binding repeats (4R tau). As the sequence corresponding to exon 10 appears to be of great importance in increasing the affinity of tau for microtubules [4], differences between 3R and 4R tau may have implications for microtubule dynamics, as well as for localization of tau to the microtubules. This is of great importance because tau with low affinity for microtubules may be released into the cytosol, where it is free to form aggregates. It is unknown whether the seeding potential of oligomers from 4R tau differs from that of 3R tau and whether this may underlie some of the differences between various tauopathies specific to the number of microtubule-binding repeats.

PSP, unlike tauopathies such as Alzheimer’s disease (AD) which form aggregates from both tau forms, has a shifted ratio of 4R:3R tau [5] and specifically forms NFTs from 4R tau [6]. Similarly, corticobasal degeneration (CBD) also exhibits aggregates from 4R tau alone [6]. However, tau isolated from CBD and PSP display different fragmentation due to variation in cleavage at the amino terminal, suggesting that the two diseases have different mechanisms of proteolytic processing for tau aggregates [7, 8]. Additionally, though most of the phosphorylation sites of misfolded tau in PSP are similar to the other tauopathies, there are some differences [9, 10]. These differences may underlie the unique pathological features and spatial organization of tau aggregation between each disease, which likely lead to differences in functional outcome. The two main pathological hallmarks of PSP include globose-type NFTs and tuft-shaped astrocytes, while other neurodegenerative tauopathies display different histological tau features [1, 11, 12]. The presence of tau aggregation in glial cells is a prominent feature in PSP. In addition to the presence of tufted astrocytes, oligodendrocytes may be affected in PSP as well. Argophylic threads and coiled bodies comprised of 4R tau have been found in oligodendrocytes in PSP cases [13]. Tau pathology is also located in different regions in PSP than in other diseases. NFTs in PSP are seen in the brainstem, basal ganglia, and the prefrontal and precentral cortex and hippocampus [12, 14]. Therefore, while tauopathies do share many common features, it is important to study the mechanism of each individually.

While NFTs are the main histological hallmark in PSP, tau, like other disease-associated amyloids, can form oligomers which then go on to form fibrils. Recent studies from our laboratory and others have shown that tau oligomers, not NFTs, are the most toxic species *in vitro* and *in vivo* and may seed the pathological spread of tau [15–18]. Tau oligomers have been shown to be present in the brains of patients with Alzheimer’s disease before NFTs can be detected [16, 19–21], correlating with dysfunction of the ubiquitin proteasome system and mitochondria [22, 23]. Moreover, cognitive and motor deficits in animal models of tauopathies correspond to levels of tau oligomers, but not to levels of NFTs [15, 24–28]. However, the role of oligomeric tau has never been investigated in PSP, though the importance of oligomeric tau in the spread of pathology in PSP has been suggested [29]. In this study, we characterized tau oligomers for the first time in human PSP brain samples and showed the potential for them to seed the oligomerization of both 3R and 4R tau, implicating oligomeric tau as an important component of PSP disease progression.

## Materials and methods

### Preparation of brain homogenate

Frozen brain tissue from the pons was attained from subjects with progressive supranuclear palsy (PSP) and age-matched control subjects from the Institute for Brain Aging and Dementia (University of California–Irvine, Irvine, California, USA) and the Brain Resource Center at Johns Hopkins. All postmortem brain tissue used were randomized. PSP tissue cases were collected with patient consent and handled under protocols approved by the Johns Hopkins Institutional Review Board. PSP samples were examined at the Division of Neuropathology at John Hopkins University. Brains were homogenized in PBS with a protease inhibitor cocktail (catalog no. 11836145001; Roche Applied Science, Indianapolis, IN, USA), using a 1:3 dilution of brain: PBS (w/v). Samples were centrifuged at 10000 rpm for 10 min at 4°C. Supernatants were aliquoted, snap-frozen, and stored at -80°C until use.

### Immunofluorescence

Sections used for fluorescent immunohistochemistry were deparaffinized, rehydrated, and blocked in normal goat serum for 1 hr and incubated overnight with anti-tau oligomer-specific polyclonal antibody, T22 (1:300). The next day, sections were washed three times for 10 min in PBS and incubated with goat anti-rabbit IgG Alexa-568 (1:500; Invitrogen) for 1 hr. Sections were again washed three times for 10 minutes in PBS and were either incubated overnight with Tau-5 (1:300) for total tau, AT8 (1:100) for Ser202/Thr205 phosphorylated tau, or PHF13 (1:250) for Ser396 phosphorylated tau. The following day, sections were washed in PBS three times for 10 minutes, then incubated with donkey anti-mouse IgG Alexa-Fluor 488 (1:500; Invitrogen). Sections were washed and incubated in DAPI (Invitrogen), then mounted using Fluoromount G (Southern Biotech) mounting medium. Sections were imaged using a Zeiss LSM 510 Meta confocal system. Six images were taken from each sample and cells were randomly selected from each image for quantification using Image-J. The total level of fluorescence was measured for each cell, as well as the level of background from three different regions around the cell without fluorescence. In order to correct the level of fluorescence for background and cell size, the background multiplied by the area of the cell was subtracted from the total fluorescence. The corrected cell fluorescence was analyzed via One-way Analysis of Variance (ANOVA).

### Western blot

Pre-cast NuPAGE 4–12% Bis-Tris Gels for SDS-PAGE (Invitrogen) were loaded with 20–25 μg of protein for each sample per well, run under reducing conditions, and then transferred to nitrocellulose membranes. Membranes were then blocked overnight at 4°C with 10% nonfat dried milk. The next day membranes were incubated with T22 (1:250) for tau oligomers, Tau-5 (1:1000) for total tau, and GAPDH (1:1000; Sigma) as a loading control, diluted in 5% nonfat dried milk for 1 hr at room temperature. Tau-5 and GAPDH immunoreactivity were detected with horseradish peroxidase-conjugated IgG anti-mouse secondary antibody (1:3000, GE Healthcare) and T22 was detected with horseradish peroxidase-conjugated IgG anti-rabbit secondary antibody (1:3000, GE Healthcare). For signal detection, ECL plus (GE Healthcare) was used. Densitometry of each band was quantified and normalized with GAPDH using Image-J and analyzed by one-way ANOVA.

### ELISA

For ELISA analysis, 96-well plates were coated with 15 μl of samples (PBS soluble fractions of brains) using 0.05 M sodium bicarbonate (pH 9.6) as the coating buffer and incubated overnight at 4°C. Plates were washed once with TBST (0.01% Tween 20), then blocked for 2 hrs at RT with 10% non-fat milk. Plates were then washed once with TBST. T22 (1:250) or Tau-5 (1:1000) diluted in 5% nonfat milk was added and allowed to react for 1 hr at RT. Plates were washed three times with TBST. T22 and Tau-5 immunoreactivity was detected using 100 μl of HRP-conjugated anti-rabbit IgG (GE Healthcare) or 100 μl of HRP-conjugated anti-mouse IgG (GE Healthcare) respectively diluted 1:3000 in 5% nonfat milk and incubated for 1 hr at RT. Lastly, plates were washed three times with TBST and incubated with 100ul of 3,3,5,5-tetramethylbenzidine (TMB-1 component substrate, from Dako) for 1 hr in the dark. To stop the reaction, 100 μl 2M HCl was applied and plates were read at 450 nm in a Polar Star Omega plate reader (BMG Labtech). Each sample was measured in triplicate and results were analyzed by student’s t-test.

### Preparation of PSP brain-derived tau oligomers (BDTO)

The immunoprecipitation of tau oligomers from PSP pons was completed as previously described [17, 30]. Thirty μL of tosyl-activated magnetic Dynabeads (Dynal Biotech, Lafayette Hill, PA) were coated with 20 μg of anti-tau oligomer-specific polyclonal antibody, T22 (1.0 mg/ml) diluted in 50 μL of 0.1 M borate, pH 9.5, keeping end concentration of beads at 20 mg/mL overnight at 37°C. Beads were washed (0.2 M Tris-HCl, 0.1% bovine serum albumin, pH 8.5) and then incubated with 100 μl of PSP brain homogenate (PBS soluble fraction) with rotation at room temperature for 1 hr. Beads were washed three times with PBS and eluted using 0.1 M glycine, pH 2.8. The pH was adjusted using 1 M Tris-HCl pH 8.0 and then fractions were centrifuged in a microcon centrifugal filter device with a molecular weight cut-off of 25 kDa (Millipore, Cat # 42415) at 14000 g for 25 min at 4°C. Oligomers were re-suspended in sterile PBS. Protein concentration was measured using the bicinchoninic acid protein assay (Pierce). The samples were then centrifuged again in a microcon centrifugal filter device with a cut-off of 25 kDa at 14000 g for 25 min at 4°C. Oligomers were characterized by various methods including size-exclusion chromatography (SEC) and atomic force microscopy (AFM) as previously described [17, 31] and stored at -80°C. Oligomers were re-suspended in PBS in order to obtain the desired concentration (0.18–1.2 mg/ml) and kept at 4°C for 15–30 min, then at room temperature for 10 min before use. Oligomers were characterized as previously described [31].

### Seeding assay

3R and 4R tau monomer were obtained by dissolving lyophilized pellets of recombinant 3R and 4R tau at 0.3 mg/mL concentration in phosphate-buffered saline (PBS) [15, 31] and seeded with PSP brain-derived tau oligomers isolated as described [17]. BDTO seeds were added at 1:100 (w/w) to 2.5 μM solutions of monomeric 3R and 4R tau in PBS (in duplicates) with gentle agitation at room temperature. Aliquots were taken at each time point and immediately added to ELISA plate wells containing coating buffer (0.05M sodium bicarbonate buffer, pH 9.6, 0.02% sodium azide). Other aliquots were added to mica sheets for AFM analysis. Samples were analyzed by AFM and ELISA using T22 and Tau-5.

## Results

### Brain sections present with tau oligomers and classical PSP histological hallmarks

**Figure 1:**
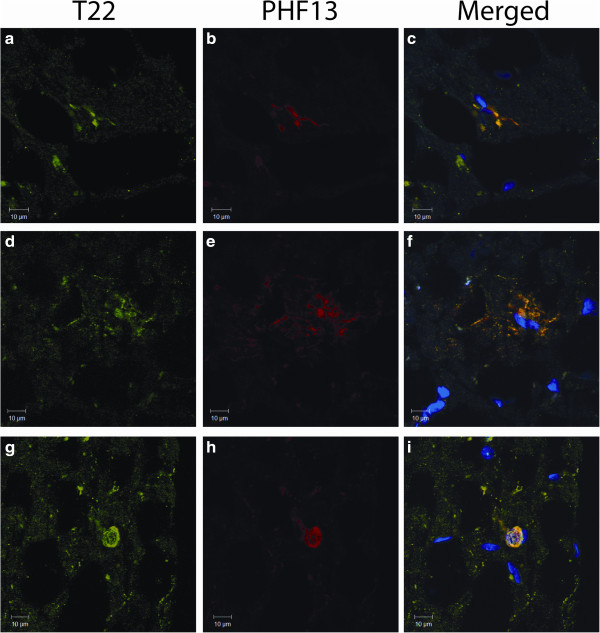
**PSP brain sections labeled with T22 and PHF13 display characteristic PSP hallmarks.** PSP brain sections immunofluorescently labeled with T22 (red) and PHF13 (green). Intracellular tau oligomers detected with T22 partially colocalize with PHF13 **(a-c)**. Oligomers resembling tufted astrocytes or extracellular deposits were also detected to partially colocalize with phosphorylated tau recognized by PHF13 **(d-f)**. Characteristic globose-type NFTs were detected by T22 and PHF13 **(g-i)**.

Sections of pons from PSP patients were labeled with T22, which is specific for tau oligomers, and with PHF13 which recognizes phosphorylated tau (Figure 1a-i). T22 was partially colocalized with PHF13 (Figure 1c, f, i), signifying the presence of both phosphorylated and unphosphorylated tau oligomers. Both extracellular and intracellular tau oligomers were recognized by T22. Tau accumulation resembling tufted astrocytes was observed to be co-labeled by T22 and PHF13 (Figure 1d-f). Globose-shaped NFTs comprised of phosphorylated tau and tau oligomers were also present (Figure 1g-i).

### Tau oligomer and NFT pathology is present in PSP brains but not in age-matched controls

**Figure 2:**
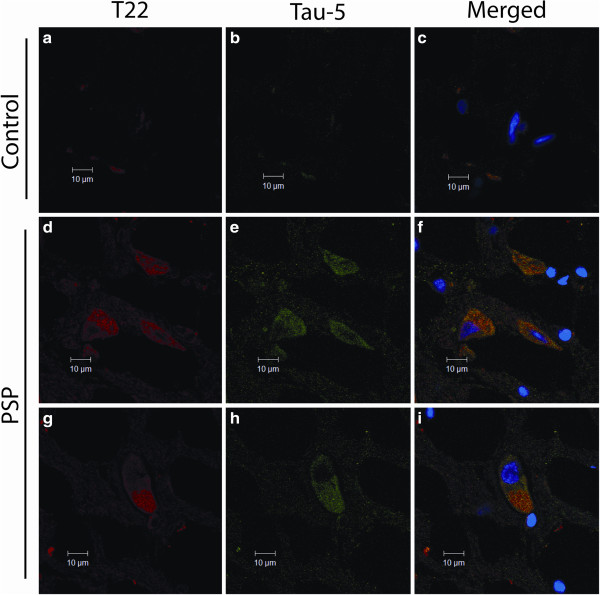
**PSP brain sections express heightened levels of tau oligomers and total tau.** PSP and age-matched control brain sections immunofluorescently labeled with T22 (red) and Tau-5 (green). Control brains do not exhibit NFTs labeled with Tau-5 or oligomeric tau **(a-c)**. Globose-type NFTs are detected with Tau-5 and partially colocalize with tau oligomers **(d-i)**.

**Figure 3:**
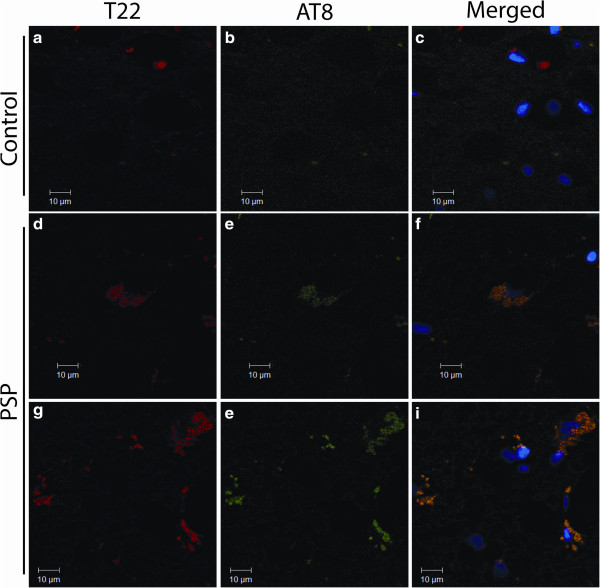
**PSP brain sections display higher levels of tau oligomer and phosphorylated tau staining.** PSP and age-matched control pons sections immunofluorescently labeled with T22 (red) and AT8 (green). Control brains do not exhibit phosphorylated tau or oligomeric tau deposition **(a-c)**. Pre-NFTs and NFTs were detected with AT8, colocalizing with T22 **(d-i)**.

Pons sections from PSP patients and age-matched controls were labeled with T22 and Tau-5 for total tau. Control brains exhibited small punctate tau staining (Figure 2a-c), while PSP brains displayed an increased amount of both tau oligomers and total tau, as well as the presence of large, globose NFTs surrounding many of the cells (Figure 2d-i). T22 showed some overlap with Tau-5, as well as some distinct oligomeric tau foci. Staining with AT8, which recognizes phosphorylated tau commonly found in NFTs, and T22 did not exhibit reactivity with control brain sections (Figure 3a-c), but showed increased levels of tau oligomers in PSP brain, as well as NFTs and pre-NFTs positive for AT8 (Figure 3d-i). T22 staining was largely colocalized with AT8, signifying the presence of phosphorylated tau oligomers (Figure 3f & i).

### Tau oligomer levels are significantly higher in PSP versus control brains

**Figure 4:**
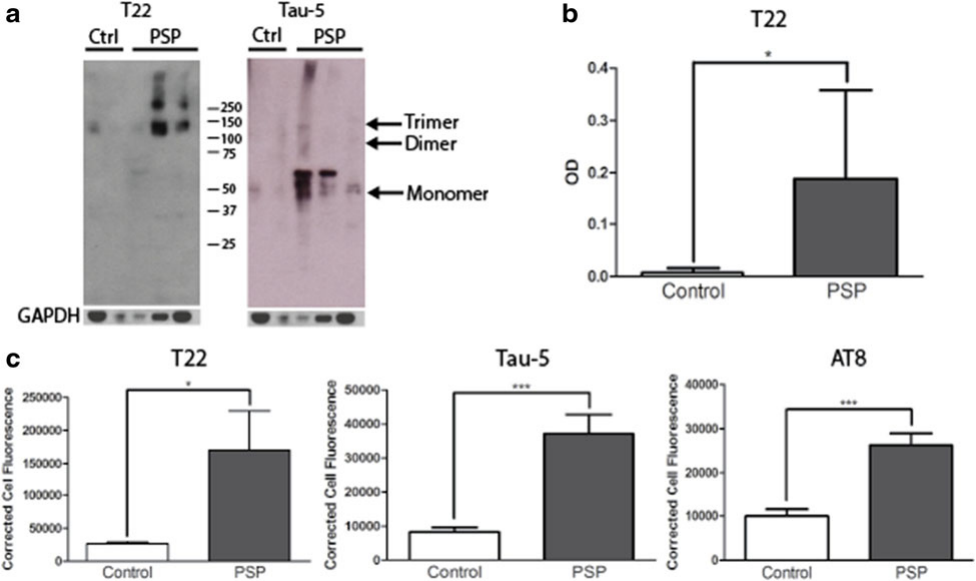
**Tau oligomers are significantly increased in PSP versus control brains.** Analysis of PSP brains via Western blot with T22 and Tau-5 showed tau oligomers, while none were detected in control brains **(a)**. Direct ELISA with T22 showed a significant increase in levels of tau oligomers in PSP patient brains compared to control brains **(b)**. Immunofluorescent staining of individual cells with T22, Tau-5, and AT8 corrected for cell size and background fluorescence showed that PSP brain samples had significantly higher levels of tau oligomers, total tau, and phosphorylated tau **(c)**. *p < 0.05; ***p < 0.001.

Analysis of PSP brains via Western blot with T22 and Tau-5 revealed the presence of high molecular weight tau oligomers (Figure 4a). Additionally, analysis by direct ELISA showed a significant increase in levels of tau oligomers detected with T22 in PSP patient brains compared to control brains (Figure 4b). Immunofluorescent staining of individual cells with T22, Tau-5, and AT8 was corrected for cell size and background fluorescence and quantified in PSP patients and controls. PSP brain samples had significantly higher levels of tau oligomers, total tau, and phosphorylated tau (Figure 4c).

### PSP brain-derived tau oligomers seed oligomerization of both 3R and 4R tau

**Figure 5:**
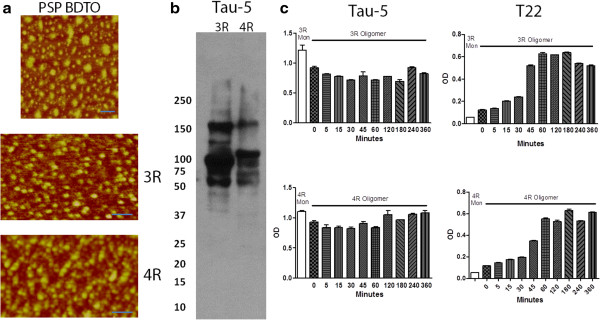
**Tau oligomers derived from PSP brain seed aggregation of 3R and 4R tau.** AFM image of tau oligomers immunoprecipitated from PSP brain and 3R and 4R tau seeded with PSP BDTO display characteristic oligomeric structure and size **(a)**. Scale bar 50 nm. Western blot with Tau-5 shows oligomeric tau present in both 3R and 4R tau seeded with PSP BDTO **(b)**. Direct ELISA with Tau-5 shows consistent tau levels in 3R and 4R tau monomer seeded with PSP BDTO and increasing levels of tau oligomers as incubation time with oligomeric tau increases **(c)**.

Tau oligomers were successfully immunoprecipitated from PSP brain, displaying characteristic oligomeric size and structure by analysis with AFM. Co-incubation of BDTO with 3R and 4R tau monomer induced the oligomerization of both isoforms of tau as shown by AFM (Figure 5a) and Western blot with Tau-5 (Figure 5b). Direct ELISA of 3R and 4R seeded with BDTO with Tau-5 confirmed consistent concentrations of tau in all samples, while ELISA with T22 displays an increase in levels of tau oligomers as incubation time with oligomers increases (Figure 5c).

## Discussion

Utilizing brain samples from the pons of PSP patients and age-matched controls, we found elevated levels of tau oligomers in PSP brains compared to controls, as well as increased total tau and phosphorylated tau in the form of NFTs. Tau oligomers were found in regions exhibiting typical PSP histological hallmarks, such as globose-shaped NFTs. The results reported here indicate that tau oligomers are important components of PSP pathology, along with NFTs. Studies supporting oligomers as the most toxic form of tau are becoming increasingly prevalent. While NFTs have been shown to correlate with disease progression in neurodegenerative tauopathies, such as AD, neuronal death and dysfunction begins to occur before the appearance of these large tau deposits [32–35]. The presence of tau annular protofibrils in glial cells in PSP as well as neurons suggests that tau oligomers may play a role in tau pathology in multiple cell types [36]. Tau oligomers lead to toxicity and cognitive deficits in mice [15] and are found to be elevated in the brains of AD patients [16, 19, 21]. However, this is the first time tau oligomers have been characterized in PSP.

Hyperphosphorylated NFTs have been known to be a main component of PSP and here we repeat results that PSP patients have increased levels of phosphorylated tau aggregates. Additionally, phosphorylated tau oligomers were detected, as well as non-phosphorylated, demonstrating the involvement of oligomeric species in the development of characteristic PSP pathology. Phosphorylation likely has important implications for toxicity, though the exact relationship between tau and phosphorylation state is not entirely certain. For one, evidence suggests that aberrant phosphorylation may increase the tendency of tau to form aggregates and that tau kinases are involved in the progression of tauopathies [37–39]. Phosphorylation state also regulates cellular localization of tau, with abnormal phosphorylation leading to the release of tau from the microtubules and redistribution from the axon to the somatodendritic compartment [40]. However, other studies have highlighted the relevance of dephosphorylated tau to toxicity and have suggested an increased ability for extracellular propagation [41, 42], implying that unphosphorylated tau oligomers detected in PSP brains may be equally important to disease progression.

The seeding of PSP brain-derived tau oligomers with both 3R and 4R tau induced the assembly of both tau isoforms into oligomers. Therefore, the mechanism by which tau pathology is induced in only 4R tau in PSP remains unknown and may not depend upon differences in tau seeding. The ability of oligomeric tau derived from PSP to seed different types of tau may underlie the frequent co-existence of other neurodegenerative tauopathies in patients with PSP [43].

There are both overlaps and differences between all of the neurodegenerative diseases, hence more research is needed to understand the exact species of tau and post-translational modifications responsible for toxicity in distinct disorders, as well as to test whether the most toxic forms of tau are also those which are responsible for the spread of disease. In spite of differences in the ratio of tau isoforms, the histological hallmarks, and the localization of tau pathology in PSP, the results reported here suggest that tau oligomers, which are both phosphorylated and unphosphorylated, are involved in PSP, similarly to AD. This implies that there is likely a common mechanism for tauopathies, similarly to other well-characterized amyloids, such as amyloid beta, whereby oligomeric species underlie toxic effects. The ability of BDTO from PSP to seed the aggregation of 3R and 4R tau monomer supports other evidence for a prion-like mechanism for the spread of tau in neurodegenerative disease [18]. These results combined with recent studies showing the efficacy of passive immunotherapy targeting tau oligomers [44, 45] support the possibility of utilizing this strategy for therapeutics in PSP, as well as in other neurodegenerative tauopathies.

## Conclusions

The results reported here show that tau oligomers are an important component of PSP pathology, similarly to what has been seen in other tauopathies, such as Alzheimer’s disease. This suggests a common mechanism for tau toxicity in pure and mixed amyloid tauopathies. Tau oligomers derived from PSP human brain tissue were capable of seeding the oligomerization of both 3R and 4R tau. As tau toxicity in PSP is primarily seen in 4R tau, rather than 3R tau, this suggests that the selection mechanism for tau toxicity may be separate from seeding. The ability of PSP-derived oligomers to seed both forms of tau also provides a potential explanation for the high prevalence of co-morbidity of additional tauopathies in patients with PSP. These results support the use of tau oligomer-directed therapeutics for the prevention of disease progression in PSP and other tauopathies.
